# Supplementary figures and images for: Associations between systemic inflammation, nutritional status, and cardiometabolic diseases and risk factors among adults living in transitional rural communities in Ecuador
Source: PLoS One. 2026 Jun 1;21(6):e0350192. doi: 10.1371/journal.pone.0350192 (PMC13225361; doi:10.1371/journal.pone.0350192)

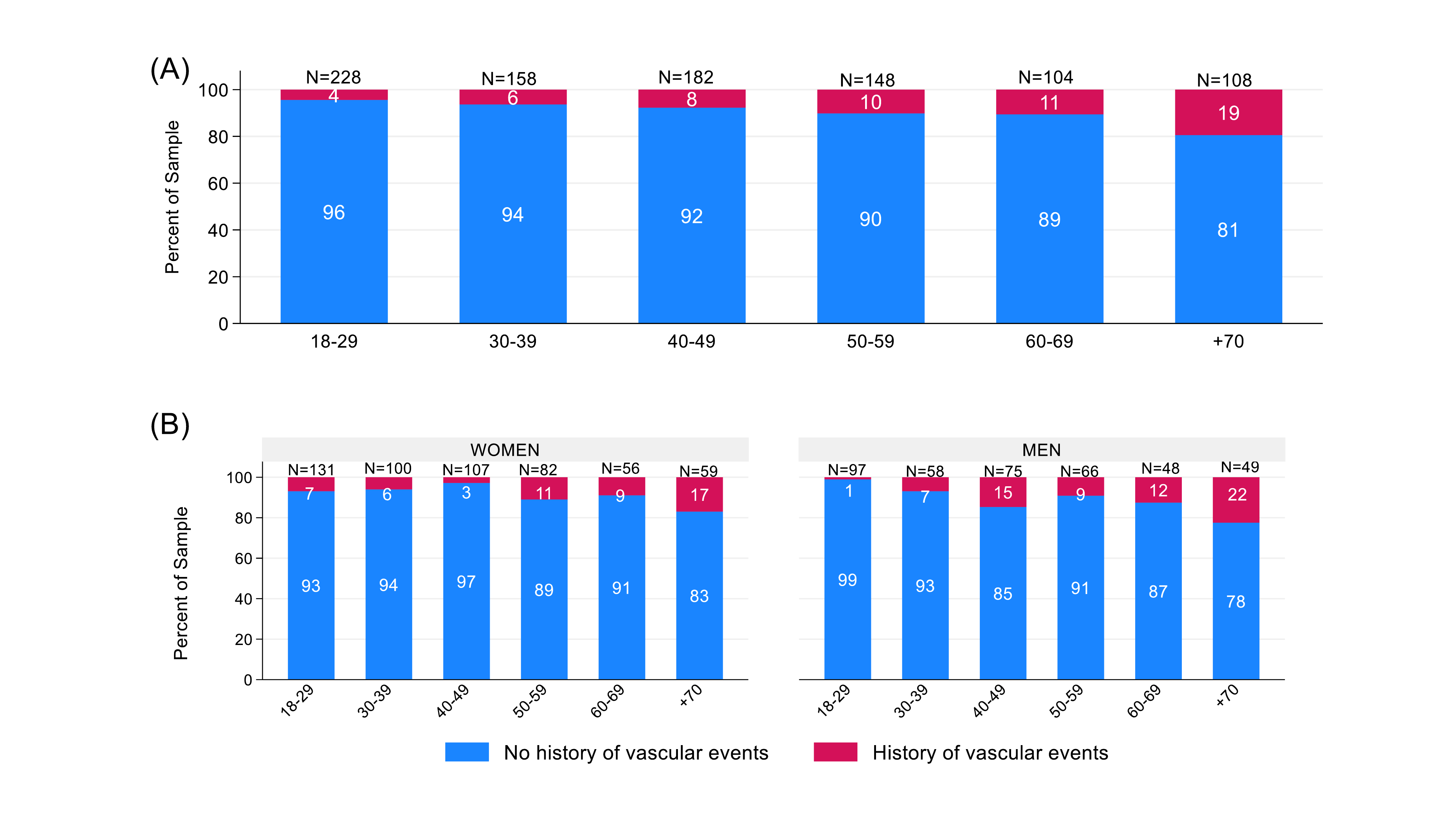

Supplement: S1 Fig — (TIFF) [file pone.0350192.s001.tiff]

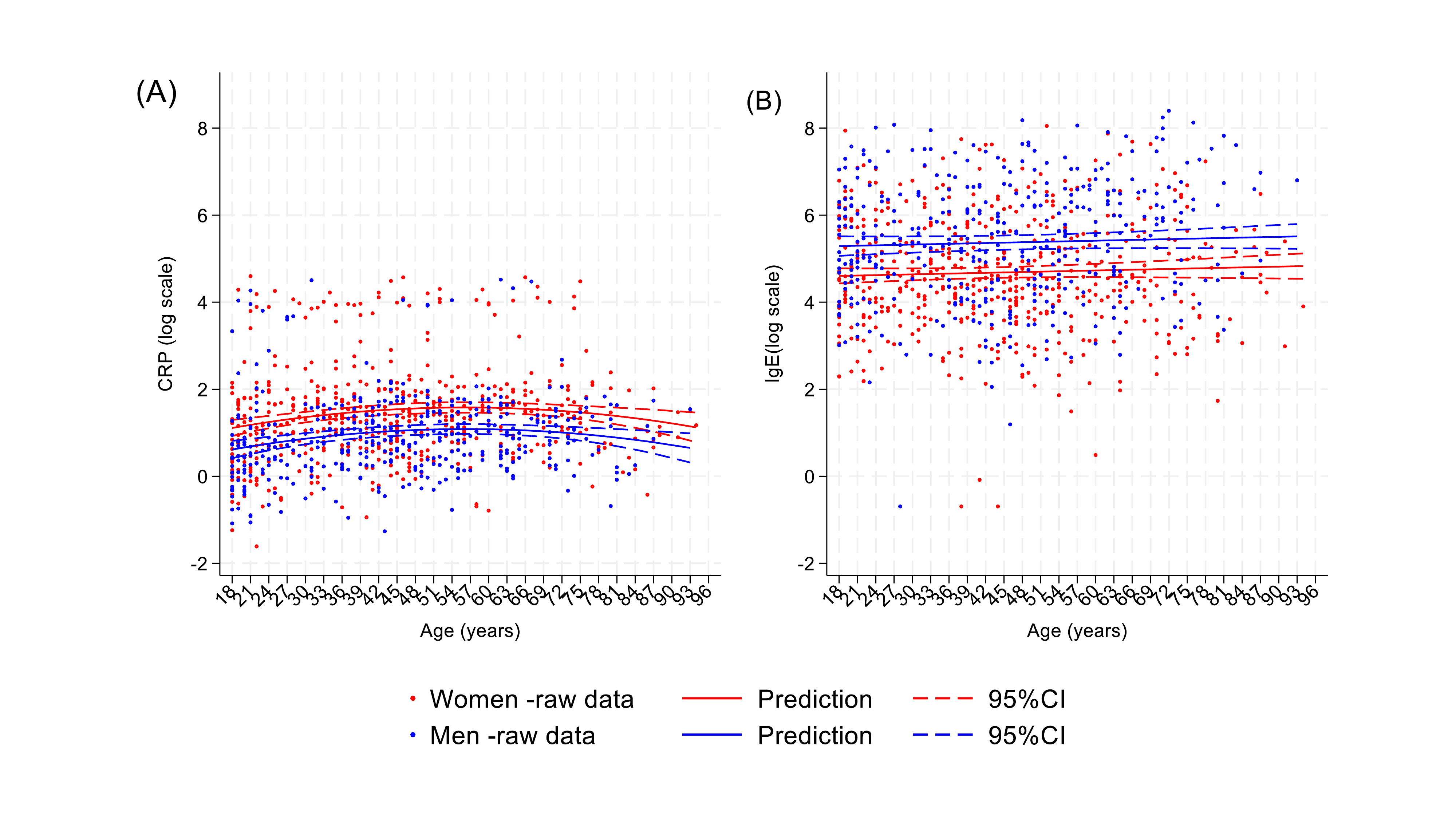

Supplement: S2 Fig — (TIFF) [file pone.0350192.s002.tiff]
